# The Clinic of Identifications in the Different Processes of Metamorphosis Into Woman

**DOI:** 10.3389/fpsyg.2018.01463

**Published:** 2018-10-22

**Authors:** Laure Westphal, Thierry Lamote

**Affiliations:** Department of Psychoanalytical Studies, Center for Research in Psychoanalysis, Medicine and Society (EA-3522), University of Paris Diderot-Paris-7, Paris, France

**Keywords:** phallic function, identification, jouissance, push-to-the-woman, psychosis, mirror stage, transsexualism

## Abstract

This article examines, from a psychoanalytical perspective, the function of identification in the relationship between the subject of the unconscious and his body, his body image, and the other. To this effect, the article leans on the clinic of the metamorphosis into a woman in psychosis, both in the way that it is presented by patients in the context of treatment, and in the form of testimonies extracted from literature. It demonstrates how specular identification allows the subject to unify himself, so long as there is an avoidance of possible deformations of the psychical body, including for example the delusion of transforming into a woman. It also turns its attention to the second logical moment of identification, when identification becomes sexed and organizes a certain relation to the other. A failure in this process sometimes leads the subject to opt for an identification of a gendered look, so as to stabilize himself. Indeed, transsexualism, which does not derive from any biological or sociological determination, and which can be observed in all subjective structures, is a possible way for the psychotic subject to problematize his relation to the body and to the other by identification with the woman, now that progress in science and law have enabled this.

## Introduction

This article looks at the function of identification as conceptualized by Lacan in the logical moments that constitute one’s subjective construction. We will look at the role of self-identification in the mirror, its stumbling in the delusion of transformation into a woman, and then the correlative pitfall of sexed identification at the moment of the Oedipus complex. Could it be possible for a subject, who lacks structuring identifications, to opt for identification with a gendered look, in order to inscribe himself into the collective?

Our development will be in four phases. First, we will show how identifications reorganize the subject’s relation to jouissance, in order to establish, secondly, the effects its failure produces on paranoid delusion. Thirdly, we will see that transsexualism is not the privilege of neurosis, since certain psychotic subjects adopt it to resolve their psychical impasse. Fourthly, and lastly, we will discuss more specifically in which conditions transsexualism represents, for the psychotic subject, a new way of inscribing himself subjectively into the collective.

## Identity, the Recognition of the Specular Image, and their Disorders Approached on the Basis of Lacanian Theory

During a group therapy session, Ferhat, a teenage patient, told us about a scene from childhood that remained deeply enigmatic to him. One day, when he was in the restaurant that his parents owned, he went to the toilet and fell into a state of stupefaction when faced with the image reflected in the mirror: “Just in front of me, in the mirror, I saw something horrible … I don’t know what it was, it didn’t look like anything I know…” He immediately slipped into a sort of somnambulism, left the restaurant, and walked for hours. What happened to Ferhat? What exactly had appeared to him in the mirror that day, in place of his specular image? Let us follow the thread of Lacan’s elaborations touching on specular recognition.

The recognition of the specular image is, according to Lacan, crucial not only for the subject’s identification – the acquisition of his identity – but also for the construction of the “reality” in which he will move. Henri Wallon was the first to pay attention to the moment when a child discovers the image of his body unified in the mirror. Moreover, Wallon shows an immediate intuition concerning the connection between body image and language. He says that specular recognition is “the prelude of the symbolic by which the mind manages to transmute what is given in sensibility into a universe” (Wallon, 1931). However, pertinent the link that Wallon intuitively establishes between image and language, we must note that he states it by inverting its terms: it is not recognition of the image that allows the symbolic function to be established, but the opposite. The prior marking of the child by the symbolic is what creates the condition not only for any recognition of his specular image, but also for the love of this body image, namely what Freud called “narcissism.” Indeed, how does the passage from autoeroticism to narcissism come about? By what modalities are the transmutation of the jouissance of the body (invaded by the disorderly chaos of the partial drives) into self-love (through the attachment of libido to the image of the body) produced? The mechanism of this tipping-point often appears very mysterious in psychoanalytical literature. Freud indicates: “There must be something added to autoerotism – a new psychical action – in order to bring about narcissism” (Freud, 1914, 77). But what “psychical action” is at work here?

In “Group Psychology and Analysis of the Ego,” Freud (1921) distinguishes identification, by which the subject enriches himself with the properties of the object, from the love bond, which, on the contrary, causes the subject to empty himself in favor of the object. Indeed, the subject’s narcissistic libido is drained by the act of love, whereas in identification, “the object becomes volatile and disappears in order to feed on the ego.” Here, the object has from the start “been lost” (Lacan, 1956–1957, 172–173). The relation to the object is established in accordance with two movements: one, identification, which is a movement of incorporation; and second, the love relation, which produces a loss in favor of the object. But these two modalities of connection to the object are, if one reads Freud attentively, conditioned by an older double operation. Such operation, he tells us, is primary, prior to the appearance of the object: the primordial identification with the father, which appears in Freud as the phase that precedes the affective bond with the mother. More precisely (in Freud, the ambiguity remains), *primordial identification* with the father and the *bond with the mother* would be almost simultaneous: they would take place in a highly condensed sequence, in a flutter. Everything happens as though identification with the father (by which the subject incorporates something of which the father is the support, namely the symbolic order) was concomitant with a loss materialized by the love bond with the mother (because in love, the subject empties himself, loses something, in favor of the other party). This sequence will be resumed and rearticulated by Freud some years later in “Negation” (*Die Verneinung*).

In ‘Negation’ (Freud, 1925), we find one of the rare Freudian articulations of primary repression and a description of the “mythical moment” (Lacan, 1956, 319) of the subject’s emergence, of the appearance of language, and of the upsurge of “reality.” Here, Freud postulates – in a phase that dates from before the distinction between inside and outside, between subjective and objective – the existence of an original “pleasure ego,” which introjects (*Bejahung*) into itself “all that is good,” while rejecting (*Ausstossung*) the bad and leaving it on the outside. The inaugural Freudian intuition, which holds that the subject’s reality is elaborated in accordance with a process of re-finding the lost object, encounters a new foundation here: the objects that constitute the subject’s reality only exist inasmuch as the primordial object has been lost. In other words, not only does subjectivity result from a fundamental loss of inside and outside, but, furthermore, it is around this lost object, whose absence will be the motor of the subject’s desire, that what we call reality will be constituted. What does *Bejahung* consist of? What is introjected at that moment, through a loss? *Bejahung*, as Jean-Claude Maleval has reminded us, is “an admission in the symbolic sense” (Maleval, 2000, 48): it is the primordial symbolization with which the symbolic order is established. This concerns the mythical moment when, through primordial identification with the father, the subject’s capture in the discourses is produced. In this moment, the subject, consenting (*Bejahung*) to inscribe himself into the order of language, accepts its essential condition, namely the loss (expulsion, *Ausstossung*) of an object, which Lacan will notate as “object *a*.” This mechanism of primordial repression, whereby the body is emptied of its jouissance (object *a*), while the subject becomes equipped with the symbolic, will likewise condition the process of specular recognition defended by Lacan in the “mirror stage” (Lacan, 1949).

We should recall that this “stage” brings the young child into presence, given his image reflected in the mirror, along with a third party (father, mother, or another) as witness to the scene. What happens during this stage? The subject, still an infant, recognizes his own image in the mirror. This recognition, Lacan tells us, must be understood as an identification, “namely, the transformation that takes place in the subject when he assumes an image” (Lacan, 1949, 76). Until then, the infant only perceived his body as scattered, disjointed elements (a hand, arm, or foot that appeared in the visual field and which he might try to grab as though it were a foreign object). Suddenly, in the mirror, what were mere fragmentations become a unified image that he recognizes as his own. At this instant, identification is produced, creating the foundation of the subject’s identity (the matrix of any speech act in the first person, “I”) through the articulation of the three registers of real, imaginary, and symbolic. Indeed, the subject knots into one unified instance the specular image, his bodily sensations (the register of the real), and the naming (symbolic) of the image reflected by the Other observing the scene. Many consequences arise from this founding moment of identity. The first is to ground the recognition (of the body image) and ego upon an error. Indeed, the bodily unity perceived in this image is falsified by specularity: the image returned by the mirror is false, inverting the body’s coordinates. Next, we must conceive how this anchors the ego in the otherness of the mirror: the ego does not coincide with itself, it stems from the register of the other (it imposes the detour via the mirror) for the subject, who finds therein the most ordinary experience of his division. Furthermore, the body image returned by the mirror locates and inscribes the locus of the other, where the ego is not only housed, but also the same others, the subject’s alter-egos (peers). Finally, the same holds for a disjunction between real and imaginary: this body that is unified and autonomous, and which is reflected by the mirror (the imaginary register), doesn’t accord with the lived experience of body incoordination, of impotence, and of *Hilflosigkeit* (the real register), where the infant is found. The image observed in the mirror is thus inscribed in the form of a developing fiction, because it anticipates a state to come in relation to the real state in which the subject is found. Here, we can see the function of the specular image: the image (i) veils, covers the real (a). Moreover, this is precisely what Lacan formalized in the *matheme* of the body image, i(a), where “i” represents the image, and “a” the real object, the lost object, rejected outside the symbolic, whose absence supports the image and ensures its consistency. Let us briefly summarize the two phases that condition the recognition of the specular image: first of all, in the initial phase, primary repression has to be produced, which allows for language access (*Bejahung*) by means of loss (*Ausstossung*) of the object (*a*). It is on the grounds of this initial loss that the mirror stage will be elaborated, the matrix of recognition (of the body image and reality), which is established through articulating specular image (i), the real (object *a*), and the symbolic (naming by the Other). The whole, as we can see, falls under the dependence on primary repression, that is, loss of the primordial object: if the lost object (the object *a*) is no longer lacking, if it reappears in the weave of the image, we will be dealing with a series of clinical phenomena that we will approach later. For now, we will look at how these two phases inscribe their effects into the subjectification process, by virtue of the retroaction phase triggered by the Oedipus complex.

The Oedipus complex confirms the loss of the primordial object and opens a resolution to the dead-end in which the subject is precipitated. We may recall that after a phase of reciprocal illusion (when the infant could derive comfort from the illusion of completing the mother, while she could also sustain this illusion of a harmony between herself and her child) the young child suspects that something is not right: the mother’s comings and goings hint toward maternal castration, indicating that she too lacks, i.e., that she desires and seems to find elsewhere (beyond the subject) the object to satisfy her desire. The child, exposed to what initially appeared as something stemming from pure whim, is then caught in an anxiety-provoking alternative, sketched out in accordance with the coordinates of his (oral) libidinal development: either he has the means to fulfill the mother, and in this case risks being devoured by her; or he lacks these means, and risks being abandoned, left in the lurch. The father’s intervention, through what Lacan named the “paternal metaphor” (Lacan, 1958, 463–4; 476; 479), traces the path of a resolution to this dead-end, which cannot be resolved in the imaginary register (where the mother/child relation is located). The Name-of-the-Father is the signifier that allows the mother’s desire to be named and for the subject to be positioned in a genealogy, that is, at a place whereby the mother is forbidden. The result of this Oedipal operation is threefold. On one hand, it inscribes the coordinates of the symbolic order (which Lacan named the Other, the treasure of the signifier), which are founded upon the law of language (the word is the death of the thing: once the object is lost, the subject only encounters its semblances through the intermediary of signifiers representing them). On the other hand – we will return to this in detail – it allows for the production of the phallic signifier, which symbolizes the loss of the object of jouissance: the phallus tallows the loss to be metabolized. Without the phallus, this loss would prove intolerable. Finally, inscribed at the resolution of the Oedipal process is what Lacan called the fundamental fantasy, which allows for the positioning of the lost object (*a*) in a certain relation to the subject (*$*): *$* ♢*a*. The fundamental fantasy is not a little fiction that the subject recounts to himself so as to be cut off from reality; on the contrary, not only will it orient the subject’s desire, but it will also provide reality with its frame. Indeed, desire is oriented by the quest for the lost object. We have seen that this is the condition of the appearance of all the objects that present themselves to the subject, in other words, all the objects around which what we call reality is arranged. Should the fundamental fantasy vacillate, reality itself careens.

Identity in Lacanian theory is, therefore, a three-phase process. In the first phase, primary repression engages the process of subjectification, placing its dependence on the loss of the primordial object; in the second phase, this inaugural loss is felt again during the mirror stage, when the lost object serves to support the specular image, covering it by connecting it to the symbolic; finally, in the third phase, the entire sequence is stabilized during the Oedipus complex, when the fundamental fantasy is established, which comes to frame and localize the object *a*. Accidents arising during one or more of the phases of this process will generate a set of phenomena touching on identity. The cause of the inability to recognize the image of the self can be located in cases of early autism (Lefort) or serious child psychosis (Maleval, 1981). These incidents arise during primary repression: when *Ausstossung* (primal expulsion) has not occurred, the specular image, un-ballasted by the lost object, is struck with a strangeness that prevents the recognition processes. How are we to tackle the passing disturbances that affect specular recognition, like when the young Ferhat saw an object of horror appearing in the mirror? What happened at that moment, in the locus of his face, was nothing other than the object *a*, without its imaginary covering and devoid of symbolic armature. In other words, the misadventure recounted by this young adolescent shows us another consequence of the foreclosure of the Name-of-the-Father: when the paternal metaphor has not been produced, the object *a*, being neither localized nor framed by the fundamental fantasy, threatens at any moment to reappear directly in the weave of reality, which it suddenly unravels, leaving the subject in a state of stupefaction. We will now turn to another series of disorders, likewise conditioned by the foreclosure of the Name-of-the-Father: the psychotic subject’s disturbances of sexed identity. Let us examine this with the case of Schreber, which will allow us to distinguish disturbances affecting the coordinates of specular recognition (implying the object *a*) from what Lacan names “push-to-the-woman” (where the question of Other jouissance arises).

## Disturbances of Sexed Identity, other Jouissance, and Transsexualism: the Example of Schreber Re-Read by Lacan

Daniel-Paul Schreber was a German jurist. In 1884, shortly after failing in the Reichstag elections, he underwent a psychotic episode that led him to the rest home of Dr. Fleichsig to be treated for hypochondria with suicidal ideation. He only spent a short time there, after which he spent “8 years […], on the whole quite happy ones, rich also in outward honors” (Schreber, 1903, 46): indeed, in the interval, he had become president of the Freiberg tribunal (1889), before exercising a mandate following his election as local representative in the constituency where he had been defeated in the 1884 elections (Devreese et al., 1986, 156). Around October 1893, when he was promoted to the presidency of the Supreme Court of the Dresden district, he had another psychotic episode, more serious this time, to the point that after some months, bombarded by multiple hallucinations, he was relieved of his functions and placed under provisional tutelage (1895). After a long efflorescent period of his delusion, he drafted his memoirs to plead his case before a tribunal. He won his lawsuit in 1902, left the asylum, and published his *Memoirs of My Nervous Illness* (1903). Some years later, in 1907, a new psychotic episode returned him to the asylum, where his state rapidly deteriorated. He died there in 1911. Freud drew on Schreber’s *Memoirs* to establish the bases of his theory of psychosis (Freud, 1910). Lacan made it the guiding thread of his 1955–1956 Seminar, *The Psychoses*, before making it the base of his own theory founded on the notion of “foreclosure of the Name-of-the-Father” in his text “On a Question Prior to Any Possible Treatment of Psychosis.”

Following the trail of Freud and Lacan, we will turn our attention to the second psychotic episode (1893). Schreber explains that when he was notified of his promotion in June 1893 by the Minister of Legal Affairs – “Dr. Schuring in person” (Schreber, 1903, 46) – he had dreams foretelling the return of his illness. One morning, he adds, a “sensation” (rather than a dream) imposed itself, striking him “as highly peculiar: it was the idea that it really must be rather pleasant to be a woman succumbing to intercourse” (Schreber, 1903, 46). He took up office in the following weeks; but faced with the heavy burden of work, he says that he quickly “overtaxed [himself] mentally” (Schreber, 1903, 47). He started to hear crackling noises, before a fresh “nervous breakdown” occurred, accompanied by troubling physical symptoms. He writes: “the blood had gone from my extremities to the heart,” while his mood was “gloomy in the extreme” (Schreber, 1903, 49). Dr. Fleichsig quickly admitted him into the care home. Schreber soon fell into catatonic states, became delusional and confused, with hallucinations. Both Freud and Lacan looked, in particular, at the initial *sensation*, which had arisen on the brink of the phenomena, that of being a woman succumbing to intercourse. For both, the entire cycle of the illness – from the virile protest at the start when first faced with this idea, through to the final reconciliation (*Versohnung*) with God, that is, with the father (Freud, 1910), via the delusional labor itself – finds its cause in this inaugural instant. Indeed, the entire delusional movement leads Schreber to rearrange reality, not to make it “more beautiful” (Freud, 1910), but simply to make it compatible with the initial intuition. According to his system, the world order had been decomposed, endangering the whole universe; the only recourse to avoid catastrophe entails putting himself in a feminine position in relation to God: once transformed into a woman, he will give birth to a new humanity, formed of the “Schreber spirit.” Only then a certain calm appeared, according to his testimony: after the terrifying experiences of the first phases – composed of invasive hallucinations, a sense of the world ending, and unspeakable bodily happenings – a respite emerged, concomitant with the acceptance of womanly transformation, allowing him to begin serenely entertaining the various procedures he would undergo to leave the asylum and put an end to tutelage. At the end of the delusional process, he maintains his conviction of transforming into a woman. He says he had observed his specular image at length: “I venture to assert flatly that anybody who sees me standing in front of a mirror with the upper part of my body naked would get the undoubted impression of a female trunk, especially when the illusion is strengthened by some feminine adornments” (Schreber, 1903, 248). What can be said of this movement toward feminization, in the way that Schreber describes it, and which in some way we feel relates to the specular image? Is it transsexualism?

In his book *Clinique de l’identité* (2009), Thibierge notes that in psychosis, the dimension of recognition or of the image proves to be fundamentally defective, to a point that, “upholding an image or a meaning always turns out to be precarious and under threat. […] This is why, in a psychosis – regardless of the apparent solidity of certain delusional edifices, where there is an attempt to suture this fault– a complete collapse of the subject’s imaginary coordinates is always liable to occur, that is, a complete collapse of what we call recognition” (Thibierge, 2009, 23). The most enlightening example of this fragility of the specular register is given by the Fregoli delusion, described by Courbon and Fail (1927). The patient of the original case said that her persecutor, the actress Robine, embodied multiple personalities, like the Italian actor Fregoli. She identified her persecutor, in disguise, in any person she met, and from whom she received “influxes” of other sensory phenomena. Robine was “always there under the variety of rags” (Thibierge, 2009, 13). In this syndrome, “the other party is always the same person,” which indicates a problem where name and image are not joined: “The name names something the image fails to cover, to represent, in short, that it doesn’t allow one to recognize: it is something else” (Thibierge, 2009, 13). Indeed, the name is no longer articulated to the image, but rather designates an object, “an *x*, which is always the same, which in coming to the fore reveals an inconsistency, even the collapse of the image and the imaginary in the field of recognition” (Thibierge, 2009, 13). This object, which shows through behind the image to the point of unjoining it from the name in order to become prevalent, is of course the object *a*, which is always at risk of appearing again in psychosis (because it is no longer framed by the fantasy). Let’s specify the three interconnected aspects of this disintegration in the coordinates of specular recognition. First, the object *a* reappears in the foreground, because the primordial rejection (*Ausstossung*) specific to primary repression hasn’t been consolidated during the retroactive phase of the Oedipus complex, for want of any paternal support. Thereafter, this prevalence of the object modifies the function of the proper name, instead of identifying the object in a differential way, that is, through mere difference from other names (without any direct link between name and object named). The name here is conjoined to the object, raising itself to the real that it names. Consequently, the Image either joins itself to the named object (allowing it to appear behind each mask), or separates itself from it during moments of fragmentation (Thibierge, 2009, 20). Within this framework, we may tackle transsexualism in the case of the psychotic subject. The psychotic transsexual claims to have a “feminine” appearance, but this appearance does not fall within the remit of the usual register of the image, which always participates loosely in semblance; what the subject targets in this naming is a being excluded from all divisions and contingencies: “Femininity is thus the name it gives to an absolutely real substance that is non-sexed” (Thibierge, 2009, 27). When he asks for his appearance and civil status to be modified, the psychotic transsexual targets an absolute identity designated by the name of *the woman*, which he has the conviction he embodies “*more really* than women” (Thibierge, 2009, 28). Thibierge writes: “Although he claims he has a feminine image and often makes this claim, [what the psychotic transsexual aims at] is much rather what he finds himself identifying in this image, which he regularly mentions when we question him: the real of a jouissance that he appeals to and sometimes experiences, a cutaneous jouissance of the envelope, the matrix, and completeness” (Thibierge, 2009, 28). This variant of the problem was observed in the Fregoli delusion, where the autotomizing and the foregrounding of the object *a* disintegrate the representational function of the proper name. The name is then joined to the object like the image, which becomes its simple mask, and is always identical. In transsexualism, the foregrounding of the object (a real, which bears a jouissance of the envelope) is joined to a name (the woman), and compels the subject to modify his appearance and his civil identity in order to adhere to the real. In the case of Schreber, are we looking at this process of the disintegrating coordinates of recognition? We think not: to grasp what distinguishes the Schreber case from cases of transsexualism in psychosis, we must relocate it through to the question of “jouissance.”

The concept of jouissance is central in Lacanian theory, so much so that it has displaced *the* question that remained unsolved for Freud: “What does a woman want?” Lacan asks instead, what would create feminine jouissance.

Freud organized his theory of libido around the question of the phallus; why is so much attention focused on the male organ? Quite simply because “the female genitals [in the child’s fantasmatic view] never seem to be discovered” (Freud, 1923, 145): sexed division of being is organized around the fact of having, or not having, a penis. On the man’s side, sexuality revolves around the organ, the penis, offering an imaginary hold on the phallic signifier, the signifier of lack, the one bearing the mark of castration. Male jouissance, which Lacan named “phallic jouissance,” is entirely subject to the laws of language, which is, sifted by castration; it is thus limited, circumscribed, and regulated. Conversely, due to the late discovery of the female organ, the unconscious lacks a signifier that would circumscribe women with a definition. Female jouissance is hence only partially ordered by the phallic signifier, that is, it only passes partially through castration. Feminine jouissance is “not all” submitted to masculine jouissance. A woman also has the possibility of gaining access to “Other jouissance,” a bodily jouissance both crazy and enigmatic. This Other jouissance is testified to in particular by mystics: since they are “not all” constrained in phallic signification, they know, in their privileged relationship with a consistent Other (God, the Beloved), ecstasies that give a glimpse of this objectless bodily jouissance by which the rift in the Other is indicated, that is, the powerlessness of words to check it. Saint Teresa and Saint John of the Cross left superb testimonies of these bodily phenomena, ranging from the most “acute” suffering to summits of ecstasy. Saint Teresa, regarding the case of a person she says she had known in this state, writes: “although of short duration, [this state] left the body absolutely broken; the pulse was so slow it seemed the soul was on the brink of being surrendered to God, no more nor less. The body loses its natural heat; but the inner fire that consumes the soul is so ardent that were it to increase just a little, God would place it at the height of its desires” (Marie-Eugène de l’Enfant-Jésus, 1988, 777). Saint John of the Cross also dealt with these phenomena of “breakings and collapses under spiritual action,” (Marie-Eugène de l’Enfant-Jésus, 1988, 777), and spoke at length of “ravishings, ecstasies, dislocations of the bones, that are always produced when the communications are not purely spiritual” (Marie-Eugène de l’Enfant-Jésus, 1988, 777). These afflictions can be followed, in accordance with regulated procedures and asceticism, by other forms of divine response. Here is how Saint Teresa describes this out-of-the-ordinary experience: “a line of fire so ardent […] I thought I might die. I didn’t know how to explain it. It is as if an invisible hand had plunged full into the fire. Ah! What fire and what sweetness at the same time. I burned with love and felt that 1 min, 1 s more, and I would not have been able to bear this ardor without dying” (Marie-Eugène de l’Enfant-Jésus, 1988, 845).

Despite the spectacular aspect of the ecstasies described by Saint John of the Cross and Saint Teresa, the feminine position they speak of remains regulated by the Name-of-the-Father, just like the masculine position: indeed, both positions are situated in relation to the phallic signifier, a heritage of the paternal metaphor. Their flights of ecstasy thus conserve a link with the symbolic order, and this is why these great mystics can testify to this, in richly descriptive metaphors. This is very different from the psychotic’s position, whose jouissance is not disciplined by the phallus, which can give rise to bodily sensations that are otherwise terrifying, and which, moreover, are very often tempered only after a long delusional labor. This is what President Schreber did, mobilizing a heavy symbolic apparatus to construct a delusion apt to raise the jouissance that was harassing him to the level of the signifier (thereby absorbing it). Henceforth, we must distinguish this modality of *push-to-the-woman* from the modality that manifests itself in the transsexual clinic.

To begin, we will note that the jouissance Schreber experienced doesn’t stem from a cutaneous jouissance, or a jouissance of the envelope, as mentioned by Stéphane Thibierge. The pivotal moments of his unmanning are inscribed in the same way as the inaugural sensation of feminine jouissance, which surprised him at the beginning of his illness: throughout his book, Schreber testifies to these bodily sensations that gave him a “definite foretaste of female sexual enjoyment in intercourse” (Schreber, 1903, 239). He explains that all the barriers that curb the ravishing and voluptuousness of men within certain limits, for him “no longer exist, indeed in a certain sense the reverse applies” (Schreber, 1903, 249). This ecstatic state sustains his illusion that the beatitudes he has passed through stem from a subjective position that was emancipated from the humiliating phallic signification that usually bounds masculine jouissance (and feminine jouissance too, albeit to a lesser extent). This ecstatic state will also lead his delusion down the path of what Lacan called “push-to-the-woman,” a feature of the ordinary clinic of psychosis, namely the spontaneous slope, for numerous “psychosed” subjects, to give body (through delusional elaborations) to *The All Woman*, that is, to a version of woman totally liberated from castration.

Likewise, the surface of his body, its superficial envelope, was not touched by the miraculous paths, but rather the real of the internal organs, the space inside the body, which is inaccessible to the gaze. Schreber describes at length, “the miracles that enacted against the organs of the thoracic and abdominal cavities.” Likewise, his conviction of being transformed into woman didn’t come from the image in the mirror, but from organic sensations, namely that “there were marked indications of an actual retraction of the male organ; frequently, however, particularly when mainly impure rays were involved, they manifested themselves in the form of a softening, approaching almost complete dissolution” (Schreber, 1903, 142). It was also the physical sensation of “a contraction of the vertebrae and possibly of my thigh bones,” and not the (visually) perceived image of the contours of his body, that convinced him of “a change in my whole stature (diminution of my body size)” (Schreber, 1903, 142). We can see that the critical period when the conviction of unmanning was established unfolded against the backdrop of the tenacious “impression” (again, physical, not specular) that his “body had become smaller by about six or eight centimeters.” (Schreber, 1903, 142).

Let us now try to discern, using Schreber’s testimony, what distinguishes the paranoiac version of the *push-to-the-woman* from the transsexual form it might take, even if not all transsexualism derives from the *push-to-the-woman*. In this article, we will limit ourselves to the clinic of psychosis. The paranoid subject, meanwhile, treats, by means of (symbolic) delusion, the invasive jouissance that perturbs the real of the organism; the imaginary, notably the imaginary that concerns their identity and bodily surface, does not play a minor role here: Schreber didn’t *see*, strictly speaking, the modifications that occurred in his body. He felt them, from the inside, and moreover he was fully conscious that the mirror (the image) couldn’t flesh out this real (which is why he disguised it: not in order to sustain a failing identity, but in order to sustain his delusion). Despite this, and contrary to transsexual psychotics, he did not change his civil status to conjoin his identity to the jouissance he felt (at no moment did he make a claim for obtaining a female identity, except at the terminal point of his delusion when he imagined himself eventually coupling with God in order to engender a new lineage). However, the psychotic subject who chooses feminine transsexualism spares himself the delirium via the modifications brought to his name (symbolic) and his appearance (imaginary).

## The Clinic of Feminine Transsexualism According to Lacan

By looking at Schreber, Lacan (1955/1956) identified the *push-to-the-woman* in its delusional version, without knowing that 20 years later, he would meet psychotic subjects in his practice suffering from this phenomenon in the form of transsexualism.

Let us reinforce that in using Lacan’s theory, we are interested in transsexualism as it appears in psychosis. In this article, we address uniquely the many forms of expression in the metamorphosis into woman, in psychosis. We can neither infer, from our observations, the subjective outcomes of transsexualism in a “normal” or neurotic condition, nor infer a biological or social existing cause, from any possible example. We cannot pathologise, on a psychological level, such a subjective choice. Consequently, in the case of psychosis, it would be inappropriate to stigmatize the transsexual phenomenon, especially when we see (we will demonstrate this mostly in the fourth part of the article) how it allows the subject to avoid the deployment of a delirium.

Let us go back to 1976, where, during his clinical presentations at the Sainte-Anne clinic, Lacan interviewed a biological man, whose psychosis left him in no doubt that he was a woman and should become one physically by means of endocrinal and surgical treatments. Even though Lacan then qualified transsexualism as pathological, he was also reworking the concepts of jouissance and identification, deepening the trench separating the delusion of metamorphosing into woman from transsexualism. Let’s turn now to the patient dubbed “Michel H.”

Michel H. was hospitalized at Sainte-Anne in 1976, after having tried to hang himself. He recalls, in speaking to Lacan, the taste he had developed in childhood for stroking and dressing in women’s clothing, beginning with his sisters’ clothes, whose femininity he envied. He would have liked to “be a girl” like them. He initially wore women’s clothes hidden from the gaze of others, saying they were “soft on his skin,” “warm on [his] body” and gave him a feeling of well-being that would have otherwise been inaccessible. He specifies that this had always given him intense satisfaction “on a sentimental level” and he counts among his qualities the fact of being “soft and gentle.” At the time of the interview, Michel H. still cross-dressed and did what he could so that his bodily appearance would convey his impression of being a woman. He shaved closely, wore makeup, and altered the texture of his skin to arouse a soft look. He modified his appearance, striving to make it more feminine by obtaining looks of approval from the Other.

But Michel H.’s insistence on living as a woman in the relation to the Other does not explain what kind of jouissance he experienced. Moreover, on the subject of his sexuality, he said that he was considerably perplexed. At twenty-two years of age, he had attempted relationships with men and women, to see which would suit him best, and concluded that neither one nor the other attracted him (Lacan, 1976, p. 314). With women, he did not feel like a man; even if penetration did procure for him a physiological pleasure that he qualifies as “masculine,” leading him to go “through to the end,” he reported that something stronger than him contradicted him, and justified him pushing his partner away. Furthermore, he tried to have sexual relations with two male childhood friends, but limited himself to timid caresses because he couldn’t manage to feel like a woman in the arms of a man. Shortly afterward, he attempted suicide.

Was the inability for him to feel that he was of the opposite sex to another partner a result of repressed homosexuality, as Freud first suggested with regard to Schreber (Freud, 1910), or did it result from a latent *push-to-the-woman* that Michel H. had not yet deciphered? In support of this second hypothesis, Michel H. said he had to negotiate the femininity that imposed itself upon him, just as much as he had to struggle against the sense of being abused. He gives an example of a time when he started to cross dress in public, and men shouted at him in the street and pushed him around. He felt he was approached in a similarly humiliating manner each time he met people who he knew; some of them would speak amongst themselves, point at him, or else try to get to know him better and go out with him. According to him, these people laughed at having unmasked him and seen that he was a man. So, he decided to stay shut at home and “disguise” himself while taking small quantities of drugs “so as to feel [his] way into the character a bit better.” By doing so, he managed to see himself as a “woman dressed as a woman,” unified and coherent, until the impression of being a transvestite man exhibiting a lie imposed itself on him again (Czermak, 1996). In these circumstances, the feeling of wanting to get rid of himself could surge up within him just as quickly, and he deflected it by breaking the mirror in which he was looking at himself. Another time, he had tried to “castrate himself” by cutting off his penis with a razor blade. He could only touch the skin due to the pain, which was too intense.

Michel H. adds to the feeling of being a woman, the sense of being objectified in relation to the Other, depersonalized and compelled to get rid of what he feels as an excess of the drive of jouissance. When he tried to castrate himself, he seemed to be responding less to a demanding paternal Ego Ideal, and more to the urge to create the lack that symbolic castration had not inscribed in him. In order to understand this, we should revisit with Lacan (Lacan, 1972–1973) the myth of *Totem and Taboo* (Freud, 1913), according to which a whole only assumes value if it is contradicted by an exception. For the neurotic, the exception is the imaginary father he has killed and whose guilt-ridden memory forces him to renounce incest, maternal jouissance, in favor of phallic jouissance. In the opposite case, in which the subject has foreclosed the symbolic father, he lacks this exception in order to subscribe to the phallic universal and to singularize himself there. He is thereby exposed to maternal jouissance, to a sardonic *push-to-the-woman* and to an anxiety of fragmentation, which can lead him to hope to remedy this through self-mutilation in order to create a lack.

But why, in order to mutilate oneself, does one choose the penis among all the other organs? At the start of life, there is nothing phallic about the penis for the subject, who takes himself rather as a whole for the Other’s phallus. Only under the threat of castration and by inscribing the signifier of the Name-of-the-Father at the heart of his subjectivity does he phallicize the penis and turn it into the signifier of desire, the phallus. In other words, the subject gives up his status as the Other’s phallus by becoming phallic himself, and by granting the phallus, beyond its status as a signified, with the status of a master signifier of the discourse about sex. By endowing it with this added value, he commits the “common error” (Lacan, 1971/1972a,b, 311) to which all subscribe. Among other effects, this phallic masquerade immunizes the subject against the perception of a sexual organ that is a disgusting deformity to be denounced as an “error of nature.” This is the case of the psychotic subject who has equally refused, along with the paternal metaphor, phallic jouissance. Such a subject experiences the penis as a part of the body even more threatening than the others, one that targets sexual jouissance and everything depersonalizing about it. To the extent that the subject has not established their distinction, he rejects the penis so as not to be taken for the phallus (of the Other) and, to this effect, he may equally emasculate himself (Donnelly-Boylen, 2016) and take the surgeon for a castrating father.

Does the subject’s attempted ordering of the invading jouissance imply a particular relation to identification? Freud underscored that the anatomical difference between the sexes had psychical consequences in the matter of sexed identification (Freud, 1905). Lacan adopted this idea and identified three logical phases that allow the subject to opt for a sexed identification (Lacan, 1973/1974). The first phase corresponds to the mythical real of the anatomical difference between the sexes that, in reality, only takes on its value in the second phase, where the subject adheres to the sexual discourse and interprets the given data with the aid of signifiers of phallic criteria. During this second stage, nature succumbs under the weight of symbolic castration and of the signifier of the phallus, allowing the subject to subjectively differentiate the sexes. Only then the third phase can take place, and the subject chooses to identify himself sexually on either the masculine or feminine side.

If the subject does not subscribe to the phallic function during the second phase of sexualization, he will not be able to refer to the phallus as the base for organizing his jouissance and choosing a sexed identification. For example, without having subjectively assented to phallic signification, Schreber (1903) was passively inhabited by the phallic signifier. He showed this through his virile protestation (Morel, 2000), which dwindled in favor of the progressive feminization of his body and his subjectivity. As for Michel H., he averted the painfulness of a body that was incoherent with his feminine experience by physically becoming a woman through gathering information on the progress of medicine. Notably, he learned that one is able to “get oneself castrated,” to “have breasts through hormonal treatment,” and to truly manage to “metamorphose into a woman.” Reinforced by these discoveries, he asked plastic surgeons to modify his face and considered similar steps in order to be castrated and to modify his secondary sexual characteristics. The idea of “taking oneself for a woman” gave him the hope of no longer experiencing the “anxieties of being a man” and of “falling out of character.” Even though Lacan said that psychoanalysis could not hope to modify Michel H.’s projects, does this mean they are deleterious to him?

The recent homologation of transsexualism in different cultures allows the psychotic subject to envisage an unprecedented way of establishing a link between jouissance and identification. Michel H. hints to this by saying that nearly all of his romantic relationships had failed except the one in which he behaved as a woman. This was his most recent attempt with a woman who also had the particularity of “admitting” that he was a woman: “I was always dressed as a woman,” he says, “even during penetration, and I felt that I was a woman during sexual intercourse.” By way of consequence, he managed to forget that he was a man and lived together with her “like two dykes.” We will conclude from this that Michel H. was suffering from a gender dysphoria that afflicted his relations with men as well as his relations with women, except when the nature of the relation allowed him to feel like a woman. Could the jouissance that was being sought out be the jouissance of the identification with the woman, and this alone? It seems so, and this identification would be existential for Michel H., who affirms that since he was very young he has lived only to be a woman. Furthermore, he says that he prefers to sacrifice his life and not have children, to have nothing but to be a woman.

When Michel H. chooses the signifier “woman” to describe his sensorial jouissance, and wishes to take on the appearance of a woman, he is different from Schreber, who elaborates a delusion in order to justify the term of “unmanning” (Schreber, 1903). On the contrary, he constructs a project of real metamorphosis and asks for authorization from society, from medicine, demanding that the insane source of the drive, his penile organ, be removed. The psychotic subject who opts for transsexualism strives to engage in the *raison d’être* of the other sex thanks to an appearance and feminine social codes with which to stabilize himself in the relation to the Other. All in all, for Schreber, the signifier “woman” justified a devastating imbalance in the drive, whereas for Michel H., it is destined to regulate and to humanize jouissance. The psychotic transsexual subject privileges the delusional imagining of the symbolic inscription of his body in the relation to the Other, which is nothing less than the definition that we give to supplementation.

Nevertheless, the primal mark of symbolic inscription in psychosis continues to have its effects. Not having singularized himself, the subject still feels himself to be the element of a whole devoid of exteriority and experiences it in the form of anxieties of fragmentation. In this way, he is able to tame his tendency toward the “push-to-exception” (Morel, 2000) and to invent singular solutions whose clothing is contingent upon an era or upon a culture. One can be, like Schreber, the woman that men are lacking, or, like the transsexual psychotic subject, the one who denounces the order of nature as not being in conformity with the being of exception that he embodies. This second eventuality allows the subject to appropriate his *push-to-the-woman* subjectively, and to make a claim for an exceptional destiny by wanting to become even more of a woman. Freud had identified and qualified psychotic feminization as asymptotic (Freud, 1910), which Lacan (Lacan, 1958, 572) took into account in the “push to” of the “push-to-the-woman” (Ménard, 2011).

The *push-to-the-woman* proceeds from a real dimension, because jouissance is attested in the body, from an imaginary dimension – which manifests itself through the subject’s fascination with the image – and even from a symbolic dimension, once one has mapped out the position of exception to which the subject aspires as an avatar of the Ego Ideal. When this clinical phenomenon is integrated into a paranoiac construction, the subject creates a new world order around the law of his being and seeks to rejoin the signifier of “woman” by means of a delusion that is proportional in intensity to the jouissance that he is seeking to absorb. Schreber exemplified this by delaying his transformation into a woman under the pressure of the delusion’s work-in-progress, until he endowed the woman that he would embody with a character that was so exceptional that he could couple with God and generate a new lineage.

As for Michel H., he explains (as other psychotic transsexuals also do), the destiny of the singular exception that he covets. After 2 weeks of hospitalization, he says he finds it hard to bear only being allowed to wear his women’s clothes at night and that this privation makes him nervous during the day, except when he dreams of his project of metamorphosis. He specifies that this consists of becoming a woman of exceptional beauty, as has been promised him by numerous scientific articles that he has read on the subject, in which a man could be much more slender, much more beautiful, and much gentler, than a true woman. Michel H. turns the signifier “woman” into the point of exception that he lacks in order to create a new order of jouissance, and thus generates the conditions in which his sexual jouissance is tamed. Lacan’s conceptualization of the knotting between Imaginary, Symbolic, and Real clarifies this even though the psychoanalyst himself was against exploiting it for the subject of transsexualism.

## Feminine Transsexualism as a Solution

Very early in his teaching, Lacan turned his attention to the dimensions of the real, the imaginary, and the symbolic that organize subjectivity. He spoke about them between the lines of his thesis on the case of Aimée (Lacan, 1932) and then reconsidered their dialectic again on the occasion of the three significant periods that marked his approach to the psychoses. In the first period, which occurred at the same time as the Seminar that he dedicated to them (Lacan, 1958), Lacan maps out how the conditions of the psychical structure and its points of fracture are determined by a psychical, logical, and linguistic causality. In psychosis, the foreclosure of the Name-of-the-Father prevents the symbolic from absorbing the imaginary. During a second period, he ventures into an analysis of Schreber’s *Memoirs* (Lacan, 1958) and puts the emphasis on the jouissance of the Other, namely the Real against which the psychotic subject can sometimes defend himself by identifying a persecutor. Lastly, he opens up a third and final period (Lacan, 1975/1976) in which he turns his attention to the way in which the psychotic subject can compensate the fragile articulation between the Real, the Imaginary, and the Symbolic.

To understand this, we may recall that at birth, the child begins by struggling against maternal jouissance, the Thing or *das Ding*, and does no more than babble a subjective claim. By turning to the father, the infant subsequently allows the Real, the Imaginary, and the Symbolic to be organized (Bousseyroux, 2011). He consolidates his subjective structuration by taking the name of his father, who names him, a step which produces a symptom at the same time. In neurosis, the symptom is thus correlative to the nomination relative to the sinthome (Lacan, 1975/1976). However, in psychosis, the subject does not have the paternal reference to constitute for himself a symptom, thus it is all the more necessary for him to fabricate a sinthome *a posteriori*, a name, in such a way that that the equilibrium of the structure will hold together. This step must circumscribe jouissance (the Real), arrange the body in the relation to the Other (the Symbolic), and exorcize the uncanny that is linked to the Imaginary. When the Name-of-the-Father is lacking, the sinthome does not replace it, but rather allows subjectivity to become organized.

After having conceptualized the sinthome, Lacan did not return to his work on transsexualism in psychosis, nor did he have any occasion to, because medicine and law were far less sympathetic to transsexuals than they have been in recent years (Bonierbale et al., 2005). In 1976, transsexualism was often considered morbid, whereas today, regardless of one’s subjective structure, one can more easily solicit medicine to modify his secondary sexual characteristics and proceed to “sex reassignment.” That is to say, a transsexual can legitimately request that the error of nature from which he suffers should be the object of reparation. A legal system has been put in place in numerous countries offering him the possibility of changing civil status and of taking a first name of the new sex to which he asserts to belong. Science and society taken as a whole have supported the project of transsexuals’. But what does this specifically allow the psychotic subject to do? In which conditions does it stabilize him or not?

Contrary to the delusion of metamorphosing into woman, feminine transsexualism (Stoller, 1968) in psychosis proceeds from the subject’s election of the signifier “woman” in order to attach the body to it. That is to say, he resolves his perplexity with regard to his body and language by overinvesting the signifier “woman” which gives the Real a meaning and offers a cartography of the drive that is more sensible. For him, this signifier performs at the same time as an advent of the body and an advent of signification (Hubert, 2007). By knotting bodily jouissance to the signifier, the subject obtains the sense of coherence that he was lacking, turning the upsetting penile into the final obstacle before reaching harmony. It is quite natural that he should thereafter request that it be cut off (Chiland, 2011).

Michel H. mentions this when he recounts a childhood nightmare in which he was terrified of a blond woman who cut off legs and bodily members in a veritable bloodbath. In order to protect himself against this woman, who “cut [also] the members of the family,” he would sleep alongside his parents in their bed. Michel H. thinks that he had discarded this nightmare by cross dressing, and yet he underlines that he has since colored his hair blond, and has also worn a blond wig. He makes a comparison between the blond woman and his own blondness. Furthermore, he remembers that this woman had aging facial features that gave her a hollowed appearance, more like a man, and that she was very unkind. He concludes that perhaps it related to the pain he inflicted on his parents by cross dressing, because in his dream, this woman always spared him by doing his parents harm. For his part, he rejects the harm that he could do to them by thinking of getting castrated far away from them, in a clinic in Morocco.

The psychotic subject often has a body that persecutes him at the level of the drive, except when he manages, as does Michel H., to turn the image perceived from the outside into the very thing that supports him. This step is uncertain, because if the Other does not consent to it, for example when the Other unmasks, mocks, or rejects the subject, the subject experiences with fright the impression of being the wasted object of the Other from which he struggles to detach himself (Czermak, 1996). He feels again the impression of having been let go, of being “fleetingly improvised” as Schreber put it (Schreber, 1903) and of risking subjective death. But if the Other authorizes him to identify himself with a woman, the subject annuls the mortifying identification with the phallus and extracts himself from the maternal jouissance. He succeeds by reaching a sensual and scopic jouissance of which he wants to be the exclusive agent. Only when he was dressed as a woman did Michel H. say his body experienced a satisfaction: “I truly find my personality, my character, and my gentleness.”

In transsexualism, where metamorphosis into woman is not delusional but requested, the subject aspires to give meaning to the excess of jouissance by qualifying it as feminine and to support himself to this effect in the eyes of the Other. The subject strives to re-knot with his ideal Ego in a pacified manner by looking at himself in the mirror that has never reflected unity. When he dresses as a woman, Michel H. says that he regains his gentleness. He completes this observation in the following way: “You can see it. My gestures are different, and my behavior, too.” Outside of him, the double being contemplates himself from the outside and becomes the person that should have been looking at him and recognizing him. Additionally, Michel H. says he has written a poem entitled “L’éternelle-La femme blonde” [“The Eternal One–The Blond Woman”] in which he describes Corinne, the female character he has forged for himself, and to whom he bears a singular veneration. He addresses her as though it were not he, but an idealized version of himself. This pacified way of living through the *schism* is possible at those moments when he manages to “forget that he’s a man” and when he makes any sense of discordance disappear behind an image that he overinvests.

But this is not all. It is not only a matter of looking like a woman (Serrano, 2007), but also a matter of being a beautiful woman, with this highly particular relation to the exception. Indeed, feminine transsexualism has the peculiarity, as opposed to its male counterpart, of worshipping this appearance in a singular fashion and of wanting to exhibit a remarkable beauty. Alone in front of the mirror and dressed as a woman, he sublimates what he perceives as a waste object, this phallus that has not been detached. With a look that is subjugated by the artifices that he models, he anticipates the body that was primarily experienced as deformed (Czermak, 2001). If he obtains a look of admiration from the Other, this represses his anxieties of fragmentation. When speaking of the blond woman, Michel H. says that he very quickly forgot that he had cross-dressed. For him, the scopic pleasure represses the obscenity of the body and allows whatever surrounds it to be invested in, in particular, the clothing that is destined to give the Ego a consistency. With the mask that he wears, the subject admires himself in the Other’s look, which subtracts the jouissance of the organ and attenuates the persecution of the look.

The scoptophilic aspect of the transsexual (Safouan, 1974) is often associated with a tendency toward exhibitionism, even to being a star. This comes from the desire to ward off the annihilating look of the Other in order to be, instead, sustained by the latter through a highlighting of the body. Etymologically, “advent” refers back to the question of dignity (Castel, 2003), which is to be underlined as central to the transsexual procedure. First, alone in front of the mirror, and then in public, the psychotic subject re-actualizes the specular experience lost to him due to not having been named at the moment of appropriating the image in the mirror. By harmonizing the body and jouissance in the Other’s look and the look from his fellow peers, he asks for their authorization so as to finally appropriate the image. He lies in wait for the look and the words of the Other to function as a unary trait, as a testimony that the egoic assumption can take place. From this perspective, losing the organ would allow everything to take shape around it. Furthermore, to be inscribed subjectively in the collective is correlative with an assumption of identity.

Ordinarily, during the specular stage, the subject not only discovers his image, but also discovers that it will not be enough to correspond with his being to the others. This is connected to a symbolic hole that is traumatic for every single subject. This pushes us to absorb the gap between these two images, and this is what the first name (Ginestet-Delbreil, 2003) facilitates by repressing the image of the body. But when the subject fails to be named, he is unable to appropriate the scopic image capable of repressing the bodily drives and must get by with a body that has not been anchored to the signifying order. Could it be that cultivating two other distinct images, whether an obscene image or a feminine image, would allow him to re-actualize the process and to provide the Other with a new opportunity to recognize him subjectively? The subject who looks at himself for the first time in the mirror has not benefitted from the assent of the Other, but it gives him the opportunity to make up for it and to validate, beyond the feminine seeming, the feminine being.

Wearing the feminine mask allows the psychotic subject to knot in a different way the dimension of the drives, the image, and the signifier “woman.” This signifier does not have the function that the signifier of the phallus holds, that of sexualizing desire, but rather the merit of differentiating the masculine and the feminine without making any appeal to the father and facilitating the subject’s subscription to a classifying identification (Morel, 2000). In this sense, a transsexual subject stripped of filiation and of origin proceeds to an auto-engendering that he would like to see validated, and chooses for himself an identification of sexed appearance that holds the value of a primordial identification. Let us underscore that this identification is the source of subjectification because the transsexual, psychotic subject who has not been able to say “my name is…” and “I’m a boy,” can now say “I’m a woman” and proudly introduce his new first name through the signifier “Mrs.” This is an experience of signifying reassignment that has a performative scope, unlike the delusion of metamorphosing into woman.

During subjective construction, the child frees himself from the gaze thanks to the name he is given, which he accepts and uses to mark out the imaginary by means of the symbolic. The transsexual follows the same path, as Michel H. demonstrates in choosing for his poem three first names in the guise of a signature: “Michel,” “Michelle,” and “Corinne.” First, he feminized his first name, then changed it for another that represented the woman he wants to become. As for his choice of the name Corinne, Michel H. explains that it refers to a childhood memory of a young girl called Corinne: “it’s a first name that I’m fond of, so I gave it to myself.” According to Ginestet-Delbreil (2003), the act of naming oneself is symbolic, because, in this way, the subject replaces the unconscious image of the body with the specular image to which he gives a meaning. The transsexual psychotic subject requests an inscription of his new first name in civil status, and re-launches the aborted process of articulating the Real and the Imaginary by means of the Symbolic.

In summary, we must distinguish, in the realm of psychosis, the delusion of metamorphosing into woman from transsexualism, where the conviction of being a woman functions for the subject as a project of inscription into the field of the Other and fellow beings. While surgery is counterindicated for the former (where the delusion needs to be contained), in the latter, it allows the transsexual subject to refuse to be the Other’s phallus by inscribing himself into the symbolic order. Indeed, the transsexual wards off the effect of his foreclosure of the phallic function with the aid of a new sexual discourse, with which he replaces sexed identification. Moreover, he defends his image in the Other’s look and supports it by means of an authentic claim for subjectivity. He knots the Symbolic to the Imaginary and no longer expects the Real of the body to confirm him by demanding his anatomical sex be modified (Millot, 1983). In this sense, the transsexual symptom functions as a supplementary device to the Name-of-the-Father, in other words, as a sinthome (Cavanagh, 2016).

Before concluding our theoretical and clinical journey on the function that feminine transsexualism takes in psychosis (for instance, the exemption of a delirium), we wish to add that we could have offered the reader a historical, anthropological, or even sociological approach to the phenomenon. However, this was not out intention, as it would have deviated from the strictly clinical aspect of this article. Psychoanalysis, based on the teachings of Lacan, must observe the choices of the subject and the outcomes of the subject’s ethics, particularly, that of not giving up his desire, obviously including when it is related to a choice of gender and/or sexual object.

## Conclusion

In the first part of this article, we proved that after the subject has gone through the logical moment of the mirror stage, he constitutes for himself an egoic identity. If, on the other hand, he does not appropriate his image with the help of an other that names him, his jouissance instrumentalizes him and the body fragments. Our re-reading of Schreber allowed us to observe that the excess of jouissance can in this context take on a feminine sense for the subject and feed a delusion of metamorphosing into woman. But, the structuralist approach (Redmond, 2013) allows us to better understand the classical delusions. This way of distinguishing the different types of jouissance, in accordance with the identification that is at stake for the subject, also sheds light on the way that transsexualism in psychosis can be presented as a solution. Indeed, not all transsexualism falls within the field of psychosis, but when it imposes itself within this subjective structure, it allows the subject who is not in the throes of the delusion of metamorphosis, but who is grappling with the jouissance of the Other, to subscribe in a different way, via the phallic function, to classifications of gender. It thus facilitates the subject’s inscription of his body and subjectivity in the collective, if the surgeon and lawmaker consent to it. Henceforth, he can sustain himself in a relation to the Other whose recognition previously failed him.

## Author Contributions

LW and TL wrote the article.

## Conflict of Interest Statement

The authors declare that the research was conducted in the absence of any commercial or financial relationships that could be construed as a potential conflict of interest. The reviewer JF and handling Editor declared their shared affiliation.

## References

[B1] Bonierbale M. Michel A. Lançon C. (2005). Le corps transformé. *Inf. Psychiatr.* 81 517–528.

[B2] Bousseyroux M. (2011). *Au Risque de la Topologie et de la Poésie. Elargir la Psychanalyse.* Toulouse: Eres.

[B3] Castel P.-H. (2003). *La Métamorphose Impensable, Essais sur le Transsexualisme et L’identité Personnelle.* Paris: Gallimard.

[B4] Cavanagh S. (2016). L. “Transsexuality as Sinthome”. *Stud. Gend. Sex.* 17 27–44. 10.1080/15240657.2016.1135681

[B5] Czermak M. (2001). *Passions de l’objet. Études Psychanalytiques des Psychoses.* Paris: Association Freudienne.

[B6] Chiland C. (2011). *Changer de Sexe, Illusion et Réalité.* Paris: Odile Jacob.

[B7] Courbon P. Fail G. (1927). Syndrome d’illusion de Frégoli et schizophrénie. *Bull. Soc. Clin. Med. Ment.* 15 121–125.

[B8] Czermak M. (1996). *Sur l’identité Sexuelle. A Propos du Transsexualisme.* Paris: L’Association Freudienne Internationale.

[B9] Devreese D. Israëls H. Quackelbeen J. (1986). *Schreber Inédit.* Paris: Seuil.

[B10] Donnelly-Boylen K. (2016). Gender dysphoria, serious mental illness, and genital self-mutilation: a case report. *J. Gay Lesbian Ment. Health* 20 376–381. 10.1080/19359705.2016.1209395

[B11] Freud S. (1905). *Three Essays on the Theory of Sexuality, in S.E*, Vol. 7 London: Hogarth, 123–245.

[B12] Freud S. (1910). *Psychoanalytical Notes on an Autobiographical Account of a Case of Paranoia S.E*, Vol. 12 London: Hogarth, 1–82.

[B13] Freud S. (1913). *Totem and Taboo in S.E*, Vol. 13 London: Hogarth, 1–161.

[B14] Freud S. (1914). *On Narcissism, in S.E*, Vol. 14 London: Hogarth, 67–102.

[B15] Freud S. (1921). *“Group Psychology and the Analysis of the Ego” in S.E*. Vol. 18 London: Hogarth, 65–144.

[B16] Freud S. (1923). *“The Infantile Genital Organization” in S.E*, Vol. 19 London: Hogarth, 139–145.

[B17] Freud S. (1925). *“Negation”, in S.E* Vol. 19 London: Hogarth, 233–239.

[B18] Ginestet-Delbreil S. (2003). *Du désaveu à l’errance. Un Préalable à la Perversion et à D’autres Phénomènes.* La Riche: Diabase.

[B19] Hubert H. (2007). Transsexualisme: du syndrome au sinthome. *Clin. Méditerr.* 76 255–270. 10.3917/cm.076.0255

[B20] Lacan J. (1932). *De la Psychose Paranoïaque Dans ses Rapports avec la Personnalité.* Paris: Seuil.

[B21] Lacan J. (1949). *“The Mirror Stage as Formative of the Function of the I,” in Écrits.* London: Tavistock, 1–7.

[B22] Lacan J. (1955/1956). *Le séminaire III, Les Psychoses.* Paris: Seuil, 1994.

[B23] Lacan J. (1956). *“Response to Jean Hyppolite’s Commentary on Freud’s ‘Verneinung’,” in Écrits in English.* New York, NY: Norton, 318–333.

[B24] Lacan J. (1958). “On a question prior to any possible treatment of psychosis,” in *Écrits: The First Complete Edition in English*, eds Miller J.-A. Fink B. (New York, NY: W.W. Norton & Company), 445–488.

[B25] Lacan J. (1976). “Entretien avec Michel H,” in *Sur L’identité Sexuelle. A Propos du Transsexualisme*, eds Czermak M. Frignet H. (Paris: AFI).

[B26] Lacan J. (1956–1957). *Le séminaire IV, La relation d’objet.* Paris: Seuil, 1994.

[B27] Lacan J. (1971/1972a). *Le séminaire XIX, …ou pire.* Paris: Seuil.

[B28] Lacan J. (1971/1972b). *Talking to Brick Walls.* Cambridge: Polity, 2017.

[B29] Lacan J. (1972–1973). *Seminar XX, Encore.* New York, NY: Norton, 1998.

[B30] Lacan J. (1973/1974). *Le séminaire XXI, Les non-dupes errent.* Paris: Seuil.

[B31] Lacan J. (1975/1976). *Seminar XXIII, The Sinthome.* Cambridge: Polity.

[B32] Maleval J.-C. (1981). *Folies Hystériques et Psychoses Dissociatives.* Paris: Payot, 2007.

[B33] Maleval J.-C. (2000). *La forclusion du Nom-du-Père.* Paris: Seuil.

[B34] Marie-Eugène de l’Enfant-Jésus P. (1988). *Je Veux Voir Dieu.* Venasque: Carmel.

[B35] Ménard A. (2011). Le pousse-à-la femme dans la psychose. *Cah. Clin. Nice* 6 133–140.

[B36] Millot C. (1983). *Horsexe, Essai sur le Transsexualisme.* Paris: Point Hors Ligne.

[B37] Morel G. (2000). *Ambigüités sexuelles. Sexuation et psychose.* Paris: Anthropos/Economica.

[B38] Redmond J. (2013). Contemporary perspectives on Lacanian theories of psychosis. *Front. Psychol.* 4:350. 10.3389/fpsyg.2013.00350 23825465 PMC3695380

[B39] Safouan M. (1974). *Etudes Sur l’Œdipe.* Paris: Seuil.

[B40] Schreber D.-P. (1903). *Memoirs of My Nervous Illness.* New York, NY: NY Review of Books, 2000.

[B41] Serrano J. (2007). *Whipping Girl: A Transsexual Woman on Sexism and the Scapegoating of Femininity.* New York, NY: Seal Press.

[B42] Stoller R. (1968). *Recherches sur L’identité Sexuelle.* Paris: Gallimard.

[B43] Thibierge S. (2009). *Clinique de L’identité.* Paris: PUF.

[B44] Wallon H. (1931). Comment se développe chez l’enfant la notion de corps propre. *J. Psychol. Norm. Pathol.* 18 705–748.

